# Supplementary material for: Parents’ and guardians’ views and experiences of accessing routine childhood vaccinations during the coronavirus (COVID-19) pandemic: A mixed methods study in England
Source: PLoS One. 2020 Dec 28;15(12):e0244049. doi: 10.1371/journal.pone.0244049 (PMC7769474; doi:10.1371/journal.pone.0244049)
Supplement: S1 File — (PDF) [file pone.0244049.s001.pdf]

Your views on routine childhood vaccination during the coronavirus (COVID-19) pandemic

INVESTIGATORS

London School of Hygiene and Tropical Medicine (LSHTM)
Sadie Bell, Pauline Paterson, Sandra Mounier-Jack

Newcastle University
Richard Clarke

INVITATION

You are invited to take part in an anonymous survey looking at parents' and guardians' views on childhood vaccination and experiences of accessing childhood vaccinations during the coronavirus (COVID-19) pandemic. We are asking parents and guardians to take part in our survey to help us identify ways of improving how childhood vaccinations are communicated and delivered during pandemics.

This research forms part of the research agenda of the National Institute for Health Research (NIHR) Health Protection Research Unit in Vaccines and Immunisation. The Health Protection Research Unit in Vaccines and Immunisation is a research partnership between the London School of Hygiene and Tropical Medicine and Public Health England, in collaboration with the University of Cambridge.

You are eligible to participate in this survey if you:

- live in England
- are aged 16 years or older
- are the parent or guardian of a child or children aged 18 months or under

ABOUT THIS SURVEY

This research has received approval from the LSHTM research ethics committee (Reference 21879)
The survey will take around 5-10 minutes to complete Your participation is completely voluntary and you do not need to answer all the questions if you do not wish to The survey is anonymous, we will not ask for any information in the

survey that could idenitfy who you are All data will be stored securely at the London School of Hygiene and Tropical Medicine Data from this survey will be made available in a public data repository for use by other researchers

AFTER THE SURVEY
At the end of the survey you will be given the opportunity to further voice your experiences and views regarding childhood vaccination during the coronavirus (COVID-19) pandemic through a telephone or Skype interview with one of the study investigators. If you decide you would like to assist further in a telephone or Skype interview, you will be asked to provide your contact details for us to get in touch with you. In this case, the contact details you provide will not be linked to any of the survey answers that you provide.

For more information about this research please feel free to contact me (Sadie Bell) at any time and I will be happy to answer your questions. You can reach me on: sadie.bell@lshtm.ac.uk. This contact e-mail (and those of the other investigators) is included again at the end of the survey.

Just here out of curiosity?
If you would like a copy of the questions we ask in this survey, please email Richard Clarke on Richard.clarke@newcastle.ac.uk.

End of Block: Introduction

Start of Block: Demographics

Sex assigned at birth

○ Male

○ Female

○ Prefer not to answer

What is your age in years?

____

In which region do you live?

- ○ South East
- ○ Greater London
- ○ North West
- ○ East of England
- ○ West Midlands
- ○ South West
- ○ Yorkshire and the Humber
- ○ East Midlands
- ○ North East
- ○ Prefer not to answer

Which of the following best describes your ethnicity?

- ○ White:- British
- ○ White:- Irish
- ○ White:- Other White background
- ○ Mixed:- White and Black Caribbean
- ○ Mixed:- White and Black African
- ○ Mixed:- White and Asian
- ○ Mixed:- Other mixed background
- ○ Black or Black British:- African
- ○ Black or Black British:- Caribbean
- ○ Black or Black British:- Any other Black background
- ○ Asian or Asian British:- Indian
- ○ Asian or Asian British:- Pakistani
- ○ Asian or Asian British:- Bangladeshi
- ○ Asian or Asian British:- Other Asian background
- ○ Chinese
- ○ Other ethnic group not represented by these options
- ○ Do not wish to say

Which of the following describe you best?

- ◯ I am raising my child(ren) with a partner
- ◯ I am a single parent
- ◯ Other
- ◯ Prefer not to answer

Which of the following best describes your employment status over the last year?

- ◯ Working full-time (over 30 hours per week)
- ◯ Working part-time (less than 30 hours per week)
- ◯ Student
- ◯ On parental leave (i.e. maternity, paternity, adoption or shared parental leave) from full-time employment
- ◯ On parental leave (i.e. maternity, paternity, adoption or shared parental leave) from part-time employment
- ◯ Combination of working full-time and on parental leave (i.e. maternity, paternity, adoption or shared parental leave)
- ◯ Combination of working part-time and on parental leave (i.e. maternity, paternity, adoption or shared parental leave)
- ◯ Homemaker
- ◯ Unemployed
- ◯ Other
- ◯ Prefer not to answer

What is your total household income in GBP (£) from all sources before tax??

- ◯ Under £15,000
- ◯ £15,000 - £24,999
- ◯ £25,000 - £34,999
- ◯ £35,000 - £44,999
- ◯ £45,000 - £54,999
- ◯ £55,000 - £64,999

- £65,000 - £74,999
- £75,000 - £84,999
- £85,000 - £94,999
- £95,000 or over
- Prefer not to answer

End of Block: Demographics

Start of Block: Main Measures

How many children are you the parent or guardian of?

- 1
- 2
- 3
- 4 or more

How many children **aged 18 months or under** are you the parent or guardian of?

- 1
- 2
- 3
- 4 or more

Display This Question:

If How many children under one year of age are you the parent or guardian of? = 1

How old is your youngest or only child?

____

Display This Question:

If How many children under one year of age are you the parent or guardian of? = 2

How old are your two youngest children?

- ▢ 1st child ____
- ▢ 2nd child ____

Display This Question:

If How many children under one year of age are you the parent or guardian of? = 3

How old are your three youngest children?

- ▢ 1st child ____
- ▢ 2nd child ____
- ▢ 3rd child ____

Display This Question:

If How many children under one year of age are you the parent or guardian of? = 4 or more

How old are your four youngest children?

- ☐ 1st child ______________________________
- ☐ 2nd child ______________________________
- ☐ 3rd child ______________________________
- ☐ Use this box for the ages of all other children

____

When is your youngest child next due a vaccination?

If you are unsure when the NHS usually recommends each vaccine, please check the following linked NHS webpage which contains all the relevant information: NHS vaccinations and when to have them

- ○ In the next 2 weeks
- ○ In 2-4 weeks
- ○ In 4-6 weeks
- ○ In 6-8 weeks
- ○ In 8-12 weeks
- ○ In more than 12 weeks
- ○ Do not know
- ○ They are overdue their next vaccination

Do you have an appointment booked for your child's next vaccination?

- Yes
- No

Display This Question:

If Do you have an appointment booked for your child's next vaccination? = Yes

How likely are you to keep this vaccination appointment?

- Very likely
- Likely
- Unlikely
- Very unlikely

Display This Question:

If Do you have an appointment booked for your child's next vaccination? = No

Have you been contacted to organise a vaccination appointment?

- Yes
- No

Display This Question:

If Has your general practice contacted you to organise a vaccination appointment? = Yes

How likely are you to accept this vaccination appointment?

- Very likely
- Likely
- Unlikely
- Very unlikely

Display This Question:

If Has your general practice contacted you to organise a vaccination appointment? = No

How likely are you to contact your general practice to organise a vaccination appointment in the near future (next 12 weeks)?

- Very likely
- Likely
- Unlikely
- Very unlikely

Please select your level of agreement with the following statements

	Strongly disagree	Somewhat disagree	Neither agree nor disagree	Somewhat agree	Strongly agree
During the coronavirus (COVID-19) pandemic, I feel it is <u>important</u> to vaccinate my child/children on time for their routine vaccinations.	○	○	○	○	○
During the coronavirus (COVID-19) pandemic, I feel it is <u>safe</u> to go to the general practice to vaccinate my child/children on time for their routine vaccinations	○	○	○	○	○
I feel that the current constraints due to the coronavirus (COVID-19) pandemic would make it difficult for me to make a vaccination appointment at my general practice	○	○	○	○	○

People who are important to me (e.g. friends, family, community etc) think that I should take my child/children to my locall GP practice for their routine vaccination during the coronavirus pandemic

- ○ Strongly disagree
- ○ Somewhat disagree
- ○ Neither agree nor disagree
- ○ Somewhat agree
- ○ Strongly agree
- ○ Do not know or have not discussed the topic

Page Break

The current recommendation from the NHS, Public Health England and the Royal College of General Practitioners is that childhood immunisation should go ahead as normal during the coronavirus (COVID-19) pandemic.

Before being contacted for this survey, were you aware of this recommendation?

- ○ Yes
- ○ No

Before being contacted for this survey, how certain were you that your child could still receive their routine vaccinations during the coronavirus (COVID-19) pandemic?

- ○ Very uncertain
- ○ Somewhat uncertain
- ○ Somewhat certain
- ○ Very certain

Have you tried to take your child for vaccinations, or tried to set up an appointment to get your child vaccinated, since the coronavirus (COVID-19) lockdown began (23rd March 2020)?

- ◯ Yes
- ◯ No

If yes,wWhen did you try to do this?

- ◯ Within the last 2 weeks
- ◯ In the last 2-4 weeks
- ◯ In the last 4-6 weeks
- ◯ Over 6 weeks ago

Display This Question:

If Have you tried to take your child for vaccinations, or tried to set up an appointment to get your... = Yes

Did you experience any issues, challenges or problems in taking your child for their vaccinations, or trying to set up an appointment to get your child vaccinated?

- ◯ Yes, there were issues/challenges/problems with this
- ◯ No, there were not any issues/challenges/problems with this

---

Display This Question:

If Did you experience any issues, challenges or problems in taking your child for their vaccinations... = Yes, there were issues/challenges/problems with this

Did you experience any of the following? Select any that apply

- ◯ GP practice closed
- ◯ Only online consultations available
- ◯ Appointment cancelled by GP practice
- ◯ Other (please state in the box provided)

____

If a new Coronavirus (COVID-19) vaccine became publicly available would you accept the vaccine for you child(ren)?

- ◯ Yes, definitely
- ◯ Unsure but leaning towards yes
- ◯ Unsure but leaning towards no
- ◯ No, definitely not

Please can you explain why?

If a new coronavirus (COVID-19) vaccine became publicly available would you accept the vaccine for yourself?

- ○ Yes, definitely
- ○ Unsure but leaning towards yes
- ○ Unsure but leaning towards no
- ○ No, definitely not

Please can you explain why?

[ ]

Do you have anything further you would like us to know about your feelings or experience of getting you child(ren) vaccination during the current coronavirus (COVID-19) pandemic? If yes, please use the text box below

- ○ I have nothing further I would like to add
- ○ [Please make use of this text box]

[ ]

End of Block: Main Measures

Start of Block: Debriefing, further recruitment

Thank you for completing this survey!

The study that you have just participated in looks at your views and experiences with routine childhood vaccination during the current coronavirus (COVID-19) pandemic.

Due to the disruption caused by the pandemic we wanted to see if parents and guardians were aware that routine vaccination programs are currently recommended as normal and how people

felt about this. The results of this study will be communicated to Public Health England and may inform future communication policy around routine vaccination during a pandemic.

Follow up telephone interview

For this study we are planning to combine the results of this survey with the findings from a range of interviews with parents of children less than 18 months of age. If you would like to help us further and would be willing to participate in an approximately 20-30 min long conversation on similar topic as that covered in this survey please provide, your email address or mobile phone details in the boxes provide below. Alternatively you can contact me directly on: sadie.bell@lshtm.ac.uk

As a thank you for taking part in interviews, participants will receive a £10 shopping voucher.
Alternatively you can contact me directly on: sadie.bell@lshtm.ac.uk

Email	
Phone	

Additional details

This study forms part of the research agenda of the Health Protection Research Unit in Vaccines and Immunisation. The primary research investigator is Sadie Bell (sadie.bell@lshtm.ac.uk). Additional researchers are Pauline Paterson (pauline.paterson@lshtm.ac.uk), Sandra Mounier-Jack (sandra.mounier-jack@lshtm.ac.uk) & Richard Clarke (richard.clarke@newcastle.ac.uk)

End of Block: Debriefing, further recruitment

Start of Block: Request for sharing with other eligible participants

Help us make the results of this project as meaningful as possible

Thank you for participating in this survey. If you have signed up for an interview, thank you and you will be hearing from one of our investigators shortly.

In the meantime, however there is a good chance that you currently know other parents/guardians with children aged 18 months or under. If you know of someone who may be eligible to take part in this survey, and think that they would like to participate, please do pass the word on about this study by sending them the link highlighted below directly.

The more people that take this survey the more meaningful the results become so any help you can give would be very much appreciated!
Link to this study: https://lshtm.qualtrics.com/jfe/form/SV_emuBZbuOBSbVNbf

End of Block: Request for sharing with other eligible participants
