# Supplementary material for: Parents’ and guardians’ views and experiences of accessing routine childhood vaccinations during the coronavirus (COVID-19) pandemic: A mixed methods study in England
Source: PLoS One. 2020 Dec 28;15(12):e0244049. doi: 10.1371/journal.pone.0244049 (PMC7769474; doi:10.1371/journal.pone.0244049)
Supplement: Attachment — Submitted filename: PLOS ONE_response to reviewers_18.11.20.docx [file pone.0244049.s002.docx]

Reviewers' comments:

Reviewer's Responses to Questions

**Comments to the Author**

1. Is the manuscript technically sound, and do the data support the conclusions?

The manuscript must describe a technically sound piece of scientific research with data that supports the conclusions. Experiments must have been conducted rigorously, with appropriate controls, replication, and sample sizes. The conclusions must be drawn appropriately based on the data presented.

Reviewer #1: Yes

Reviewer #2: Yes

2. Has the statistical analysis been performed appropriately and rigorously?

Reviewer #1: Yes

Reviewer #2: Yes

3. Have the authors made all data underlying the findings in their manuscript fully available?

The [PLOS Data policy](http://protect-eu.mimecast.com/s/qNfGCj814Cjy8rGf13uBt?domain=plosone.org) requires authors to make all data underlying the findings described in their manuscript fully available without restriction, with rare exception (please refer to the Data Availability Statement in the manuscript PDF file). The data should be provided as part of the manuscript or its supporting information, or deposited to a public repository. For example, in addition to summary statistics, the data points behind means, medians and variance measures should be available. If there are restrictions on publicly sharing data—e.g. participant privacy or use of data from a third party—those must be specified.

Reviewer #1: No

The full dataset is stored in a data repository: LSHTM Data compass. Data are available to bona fide researchers upon request and agreement by the study team. Data cannot be shared without restriction as study participants agreed that their anonymised survey and interview data could be accessed by other researchers only. This level of data access was approved by the LSTHM Observational Research Ethics committee (study reference: 21879).

Further information on the data and access conditions can be found through the LSHTM Data Compass at: https://doi.org/10.17037/DATA.00001861. Researchers interested in accessing the data are advised to request access through the LSHTM Data Compass page listed above, email the corresponding author or email researchdatamanagement@lshtm.ac.uk

Reviewer #2: Yes

4. Is the manuscript presented in an intelligible fashion and written in standard English?

PLOS ONE does not copyedit accepted manuscripts, so the language in submitted articles must be clear, correct, and unambiguous. Any typographical or grammatical errors should be corrected at revision, so please note any specific errors here.

Reviewer #1: Yes

Reviewer #2: Yes

5. Review Comments to the Author

Please use the space provided to explain your answers to the questions above. You may also include additional comments for the author, including concerns about dual publication, research ethics, or publication ethics. (Please upload your review as an attachment if it exceeds 20,000 characters)

Reviewer #1: This is a comprehensive and interesting study about exploring the experiences of routine childhood vaccination in the midst of COVID-19 pandemic. This is an important area of study given the extensive data form diverse settings.

The manuscript is written comprehensively and research design is appropriate with proper integration of quantitative and qualitative findings. Exploring also the aspects of healthcare providers for vaccination during pandemic will provide complete picture of lessons and programmatic implications in improving vaccination services during pandemic situation. Overall, the study provides valuable insights for barriers in uptake immunization services during pandemic situation with high applicability for local and global context. The comments were shown below.

Thank you so much to reviewer 1 for taking the time to read and review our manuscript.

Introduction

•The problem is properly introduced and the justification is robust enough to support the objective of the study.

Methods

•Line 97: Why the study included only 18 months or under children although GP surgery provided immunization up to more than 3 years old?

- During the COVID-19 pandemic, the priority for vaccination services is to maintain delivery of vaccinations for babies, infants and pre-school children.
- The recruitment for our study specifically focused on parents and guardians with children aged 18 months or under as most childhood vaccinations delivered in general practice are due before a child reaches 18 months of age (i.e. vaccinations are scheduled at 8 weeks, 12, weeks, 16 weeks and 12 months). There is then a gap in when routine childhood vaccinations are due until a child reaches 3 years and 4 months.
- As we wanted to find out about parents and guardians views and experiences of accessing routine childhood vaccinations we chose to focus on this time period (18 months or under) as one in which children would most likely be due a vaccination.
- The inclusion criteria were discussed with immunisation representatives at Public Health England.

We have added the following to the manuscript, in lines 97-104:

‘Our study specifically focused on parents and guardians with children aged 18 months or under as most childhood vaccinations delivered in general practice in the UK are due before a child reaches 18 months of age (i.e. vaccinations are scheduled at 8 weeks, 12 weeks, 16 weeks and 12 months). There is then a gap in when routine childhood vaccinations are due until a child reaches the age of 3 years and 4 months. Including children up to 18 months meant we could capture the views and experiences of parents and guardians whose children may have been overdue their 12 month vaccinations.’

•Line 143: What about the association between awareness of routine vaccination with the gender and education status of parents or guardians?

Thank you. These would have been interesting to examine. In designing the study, we chose to ask about location, ethnicity, employment status, household income, and number of children. An exploration of gender as an influencing factor in the study was not possible due to the limited number of men completing the survey, and we did not collect data on additional demographic factors that may have been of interest, such as education level, health literacy and religious beliefs.

A note referring to this is now included in the discussion section (lines 487-490)

•In recruiting and collecting data for semi-structured interview, there is no information about characteristics of interviewers and saturation of data.

Lines 178-180: ‘Interviews were conducted between 27th April and 27th May 2020 by SB and PP, qualitative researchers who have extensive experience in conducting interviews on the topic of vaccination with parents.’

Through the analysis of free-text responses and interviews the authors feel that we achieved data saturation, as no new themes were emerging from open-text or interview responses.

•Line 165: Is it online based written consent?
We have added the following to lines 173-174: ‘Written informed consent was obtained from each participant. Depending on the preference of the participant, the consent form was sent and returned via email or post.’

•Line 169 & 174: What are meanings of abbreviations; SB, PP, RC and SM-J?
These are the initials of the study researchers.

•Line 185 & 198: Please check appropriate reference for “Error! Reference Source not found”

Thank you. We have addressed these error messages – which seem to have occurred as the paper was converted from word document to a PDF when uploaded to the journal.

•Line 194 – 197: The selection criteria for choosing 61 parents among 530 with detail contact information is not clear. Only 19 out of 61 invited (31%) were interviewed which might lead to non-response bias.

Our recruitment approach is outlined in lines 166-172:

‘Respondents who had left their details were purposively contacted based on a range of characteristics, including ethnicity, household income, and geographical location. We also purposefully aimed to interview survey participants who did not provide free-text responses and respondents whose children were overdue a vaccination, or due a vaccination within 4 weeks of taking part in the survey.’

And we have added the following to the limitations section, lines 476-479:

‘A limitation of the interviews was that only 19 of 61 invited respondents took part, which may have been partly due to respondents not seeing the invitation email or not having the time to participate. It may be that those that took part in interviews had a stronger interest in vaccinations and a different view to those that did not take part.’

•In table 2: There are only 2 respondents whose children were overdue a vaccination. It is questionable whether the data is saturated enough in exploring the barriers of taking vaccination among such group.
Interviews were conducted as part of the study to add to the data collected through the survey. As part of the survey, rich and detailed information was obtained in open-text responses and findings from the interviews supplemented this. Through the analysis of open-text responses and interviews the authors feel that we achieved data saturation, as no new themes were emerging from open-text or interview responses.

We have added this to the strengths section of the paper (lines 471-475):

‘Using a mixed-methods approach in this study allowed for a more complete insight into parents’ and guardians’ views and experiences of vaccinating young children during the COVID-19 pandemic in England than could have been achieved using one method only. As part of the survey, rich and detailed information was obtained in free-text responses and findings from the interviews supplemented this.’

Finding

•Table 5: Why medium income level and age group 6-8 months were taken as reference in logistic model?

For the income groups the data was split into quartiles and the lower and upper qualities were compared to the middle group so as to capture differences across income. Similarly, the middle age group was taken so as to better compare between those with a high number of vaccines typically scheduled (early infancy and around 12 months) and a group where vaccines are less often scheduled.

•Line 237 – 247: The order of factors interpreted in text is better to follow the order as shown in table.

Thank you. We have changed the order of the factors interpreted in the text to reflect the order of the table.

•Line 248 – 264: It would be better to include sub-heading for these paragraphs as these paragraphs provide information not directly linked with factors associated with lack of awareness.

Thank you. This paragraph describes in more detail factors associated with lack of awareness of vaccination service continuation. We have clarified this in the text.

•Line 268: Please check appropriate reference for “Error! Reference Source not found”

Thank you. We have addressed these error messages – which seem to have occurred as the paper was converted from word document to a PDF when uploaded to the journal.

•Line 369: Who or which organization sent confusing text-messages?

Thank you. We have now clarified this to say (lines 386-387)

‘Several interview participants also discussed receiving confusing text-messages about their appointment **from their GP practice** that stated their appointments had been cancelled and they should not attend the GP practice’.

Discussion and conclusion

•Although the scope of the study is big, the discussion provides solid messages to support the conclusion. In presenting discussion, it would be better to follow the sequence of information; capability, motivation and opportunity as written in results part for better linkage.

Thank you. We have further reflected on the order of the discussion, and have kept the current flow of information as we think that it best highlights the key issues and keeps the word count down.

Reviewer #2: This is well-organized/planned survey for the effects of pandemic on routine immunization for infants below 18 months. This is very important point for ongoing COVID-19 pandemic (potential further wave) or other pandemics in future.

Thank you so much to reviewer 2. We appreciate you taking the time to read and review our manuscript.

Authors clearly defined their aims and methods using prompt references.

There are some minor points (maybe limitations)

- Number of interviewed participants is low; only 19. Regarding enrolment number, it is difficult to evaluate this small group response.

The interviews were conducted to supplement the survey findings, which included detailed and lengthy open-text responses, and provided a more comprehensive understanding of parents’ views and experiences. We purposively invited participants with less detailed open text responses for interview. The authors feel that due to the rich information obtained in open-text survey responses and the interviews we achieved a stage where no new themes were emerging from the data.

- Your title includes terms "parents", however as usual for studies during infancy, 90%of responses came from mothers. This is not parent's view, this is mother's perspective.

Thank you for your comment. On reflection, we agree that it is a limitation that more male participants did not take part in the study; however, the study was not just open to female participants (and some male participants did take part). We therefore want to keep the proposed title and have added to the limitations section that few male parents and guardians participated.

- Table 4-5 (coloured-component of COM-B) is busy to follow-up, maybe move to supplementary file or need to re-organize.
We have further reflected on the layout and location of the tables, and have kept the tables within the main text as we think they are best placed here. We have condensed the quotations in table 4 to reduce the size of the table and make the table easier to read.

- I am not sure the Journal Policy (page limitations), your Results section is too long and difficult to-follow up.

Thank you. We have shortened and changed the order of the Results section to be clearer. The length of the manuscripts fits within the journal requirements.
